# Functional domains of the FSHD-associated DUX4 protein

**DOI:** 10.1242/bio.033977

**Published:** 2018-04-04

**Authors:** Hiroaki Mitsuhashi, Satoshi Ishimaru, Sachiko Homma, Bryant Yu, Yuki Honma, Mary Lou Beermann, Jeffrey Boone Miller

**Affiliations:** 1 Department of Applied Biochemistry, School of Engineering, Tokai University, Kanagawa 259-1207, Japan; 2 Department of Neurology, Boston University School of Medicine, Boston, MA 02118, USA

**Keywords:** DUX4, Facioscapulohumeral dystrophy, Homeodomains, Muscular dystrophy, Skeletal muscle, Transactivation domain

## Abstract

Aberrant expression of the full-length isoform of DUX4 (DUX4-FL) appears to underlie pathogenesis in facioscapulohumeral muscular dystrophy (FSHD). DUX4-FL is a transcription factor and ectopic expression of DUX4-FL is toxic to most cells. Previous studies showed that DUX4-FL-induced pathology requires intact homeodomains and that transcriptional activation required the C-terminal region. In this study, we further examined the functional domains of DUX4 by generating mutant, deletion, and fusion variants of DUX4. We compared each construct to DUX4-FL for (i) activation of a DUX4 promoter reporter, (ii) expression of the DUX4-FL target gene *ZSCAN4*, (iii) effect on cell viability, (iv) activation of endogenous caspases, and (v) level of protein ubiquitination. Each construct produced a similarly sized effect (or lack of effect) in each assay. Thus, the ability to activate transcription determined the extent of change in multiple molecular and cellular properties that may be relevant to FSHD pathology. Transcriptional activity was mediated by the C-terminal 80 amino acids of DUX4-FL, with most activity located in the C-terminal 20 amino acids. We also found that non-toxic constructs with both homeodomains intact could act as inhibitors of DUX4-FL transcriptional activation, likely due to competition for promoter sites.

This article has an associated First Person interview with the first author of the paper.

## Abstract (precis)

**Summary:** Aberrant expression of DUX4 underlies facioscapulohumeral muscular dystrophy. This study identified functional domains of DUX4 and demonstrated that multiple pathological changes are related to DUX4-mediated transcriptional activation.

## INTRODUCTION

Aberrant expression of the full-length isoform of the double homeobox protein DUX4 (DUX4-FL), particularly in skeletal muscle, appears to underlie pathogenesis in facioscapulohumeral muscular dystrophy (FSHD). In FSHD, the 424 amino acid DUX4-FL protein is expressed from an open reading frame in the most telomeric 3.3 kb D4Z4 repeat on chromosome 4q (Lemmers et al., 2010). In cultures of myogenic cells or iPS cells from FSHD patients, DUX4-FL expression from its endogenous promoter is detectable by immunocytochemistry in only a small percentage of nuclei in differentiated myotubes (Haynes et al., 2017; Himeda et al., 2014; Homma et al., 2015; Jones et al., 2012; Snider et al., 2010). Aberrant expression of DUX4-FL in FSHD is associated with a decreased number D4Z4 repeats, DNA hypomethylation, and a telomeric sequence that is used as a poly-adenylation signal for the DUX4-FL mRNA (Daxinger et al., 2015; Gatica and Rosa, 2016; Hewitt, 2015; Himeda et al., 2015; Tawil et al., 2014; Wang and Tawil, 2016). DUX4-FL is a transcription factor, and ectopic expression of DUX4-FL can induce aberrant gene expression patterns and cellular pathology, including cell death, even when expressed at a low level (Bosnakovski et al., 2008b, 2017b; Jones and Jones, 2018; Kowaljow et al., 2007; Mitsuhashi et al., 2013). A shorter DUX4 isoform (DUX4-S) that consists of just the N-terminal 159 amino acids (including both homeodomains) of DUX4-FL is not toxic (Geng et al., 2011).

In addition to altering the skeletal muscle transcriptome, endogenous or exogenous expression of DUX4-FL induces multiple changes in cellular and molecular properties that may be linked to FSHD pathology. For example, DUX4-FL alters splicing patterns, as well as expression, of multiple genes (Banerji et al., 2017; Jagannathan et al., 2016; Rickard et al., 2015). In addition, DUX4-FL expression alters proteostasis and induces nuclear aggregation of TDP-43, FUS, and SC35 (Homma et al., 2015, 2016); leads to accumulation of dsRNA and nuclear aggregation of EIF4A3 (Shadle et al., 2017); and inhibits nonsense-mediated decay (Feng et al., 2015). Previous studies of DUX4-FL structural domains have identified amino acid sequences that mediate nuclear localization (Corona et al., 2013) and have shown that DUX4-FL-induced cytotoxicity requires intact homeodomains and a transcription-activating domain (TAD) in the C-terminal region of the protein (Bosnakovski et al., 2008a, 2017a; Choi et al., 2016b; Corona et al., 2013; Geng et al., 2012; Mitsuhashi et al., 2013).

In this study, we further examined the functional domains of DUX4 by generating a series of plasmids to express a new collection of mutated, deletion, and fusion variants of DUX4. We compared these constructs to DUX4-FL for (i) ability to activate a DUX4 promoter reporter; (ii) expression of the DUX4-FL target gene *ZSCAN4* mRNA (Yao et al., 2014); (iii) activation of endogenous caspases; (iv) effect on cell viability; and (v) protein ubiquitination (Homma et al., 2015). These studies showed that the extent of each indicator of cellular and molecular pathology was closely correlated with the transcriptional activating ability of each construct. In addition, the extent of transcriptional activation was determined, in large part, by the most C-terminal 20 amino acids (405-424), with a small contribution from a domain within amino acids 344-404. We also showed that those constructs that had both homeodomains intact and were non-toxic in the other assays could inhibit DUX4-FL in the promoter assay, suggesting that inhibition was likely due to competition for promoter sites.

## RESULTS

**Fig. 1.**
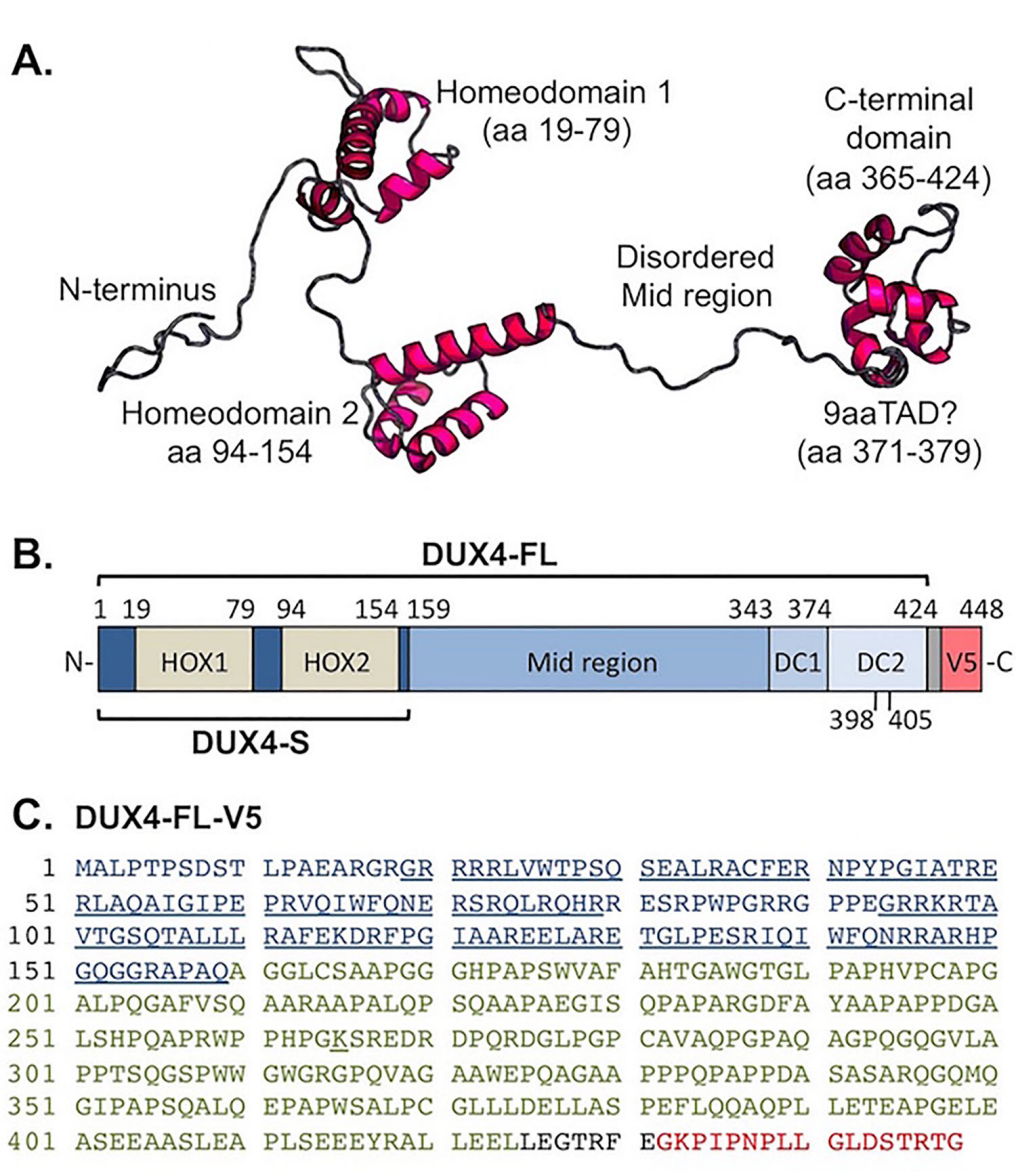
**The DUX4 protein.** (A) Ordered and disordered regions in the DUX4-FL protein as predicted by RaptorX Structure Prediction (raptorx.uchicago.edu). The two DNA-binding homeodomains and a C-terminal were predicted to have defined tertiary structures, whereas the ‘Mid’ region between homeodomain 2 and the C-terminal was predicted to be disordered. Shown is the most likely of the many similar structures returned by RaptorX. Similar predictions of ordered and disordered domains were generated by other prediction sites (not shown) as described in the Materials and Methods. In addition, there is a potential nine-amino acid transcription-activating domain (9aaTAD) at amino acids 371-379 as predicted by the online Nine Amino Acids Transactivation Domain Prediction Tool (http://www.med.muni.cz/9aaTAD/). (B) Linear representation of the DUX4 protein and sites of modification for this study. The diagram shows the two homeodomains, the predicted disordered Mid region, and sub-regions of the C-terminal domain as used to generate the DUX4 deletion and fusion cDNA constructs that are listed in Table 1. Each construct was modified by addition to the C-terminus of a seven-amino acid linker (gray unlabeled box) and the 17-amino acid V5 epitope. (C) Amino acid sequence of the full-length DUX4-FL-V5 protein as expressed in this study. The first 159 amino acids that compose the DUX4-S isoform are shown in blue with the two homeodomains underlined. The remaining amino acids (160-424) of endogenous DUX4-FL are shown in green, the linker sequence is in black, and the V5 epitope is in red.

Based on previous studies and use of the RaptorX algorithm (Källberg et al., 2012) for 3D structure prediction (Fig. 1A), the endogenous DUX4-FL protein was expected to have well-defined tertiary structures in each of the two DNA-binding homeodomains (amino acids 19-79 and 94-154) and in the most C-terminal region (amino acids ∼365-424). The C-terminal region includes the TAD and a p300 binding domain (Bosnakovski et al., 2008a, 2017a; Choi et al., 2016b; Corona et al., 2013; Geng et al., 2012). In contrast, the region between the second homeodomain and the C-terminal domain (amino acids ∼155-364) was consistently predicted to be disordered by multiple prediction sites (Fig. 1A and not shown, see Materials and Methods). In addition, there was a potential nine amino acid transcription-activating domain (9aaTAD) at amino acids 371-379 (classified as a 92% match). With this understanding of the structural and functional domains of DUX4-FL (Fig. 1B), we constructed a series of deletion, mutation, and fusion cDNA constructs (Table 1) to further probe DUX4 domains. Each construct was modified by addition to the C-terminus of a seven amino acid linker and the 17 amino acid V5 epitope tag for immunodetection (Fig. 1B,C).

**Table 1.**
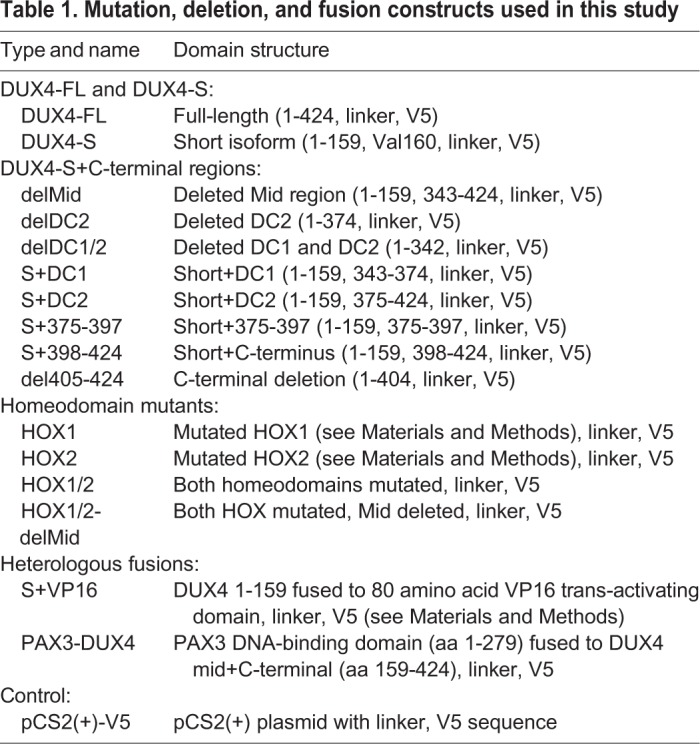
**Mutation, deletion, and fusion constructs used in this study**

**Fig. 2.**
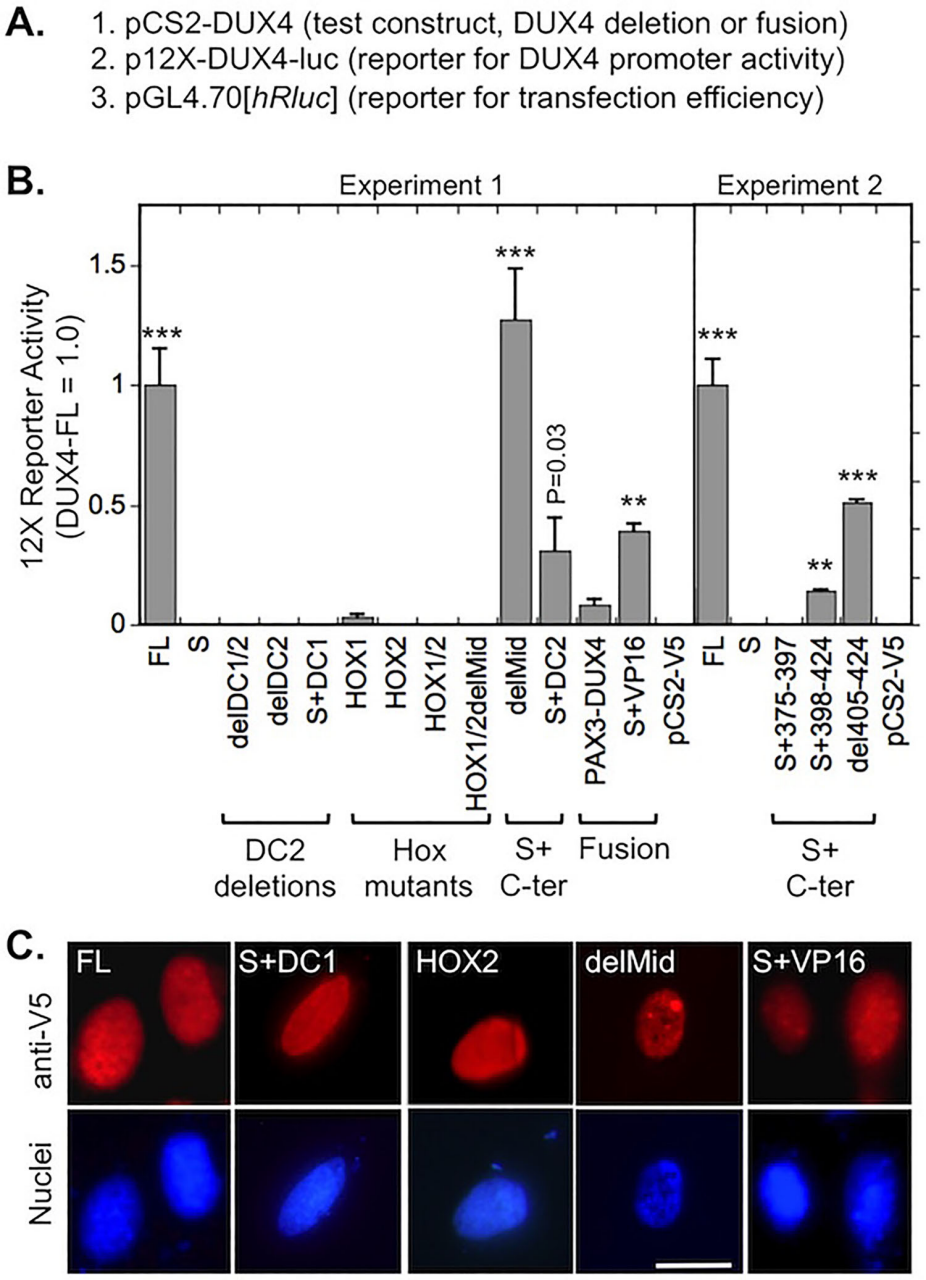
**Activation of the 12X-DUX4 promoter-Luciferase reporter by DUX4 deletion and fusion constructs.** (A) For this experiment, three plasmids were co-transfected into HEK293 cells including (i) the DUX4 deletion or fusion construct that was to be tested for activation of the 12X reporter, (ii) the 12X-DUX4 promoter-Luciferase reporter to measure DUX4 promoter binding and activation of the luciferase reporter gene, and (iii) a *Renilla* luciferase reporter to measure transfection efficiency for use in normalization. (B) Activation of the p12X-DUX4-luc reporter by DUX4 deletion and fusion constructs (see Fig. 1B and Table 1 for details of constructs). The 12X reporter was activated by intact DUX4-FL (FL) and, to varying extents, by protein constructs in which DUX4-S was fused with C-terminal sequences from the DC2 region (S+C-ter). In contrast, the 12X reporter was not activated by DUX4-S (S), by constructs lacking a TAD due to deletion of all or the most C-terminal amino acids of the DC2 region (DC2 deletions), or by mutations in one or both homeodomains (Hox mutants). For the fusion constructs, the 12X promoter was activated by DUX4-S-VP16 TAD (S+VP16) and to a lesser extent by PAX3-DUX4. ***P<0.001, **P<0.01; compared to vector control (pCS2-V5) by Dunnett method; means±s.e.; *n*=3 in both experiments. (C) All modified DUX4-V5 proteins were localized to nuclei. Five examples are shown, including three that activated the 12X reporter (FL, delMid, S+VP16) and two that did not activate the 12X promoter (S+DC1, HOX2), but all constructs used here showed similar nuclear localization. Scale bar: 20 µM.

We first examined to what extent each of the DUX4 constructs was able to activate the DUX4 promoter when expressed in HEK293 cells. For this study, we used the sensitive promoter activity assay method developed by Zhang et al. (2016), which uses a 12X multimer of DUX4 binding sites coupled to a luciferase reporter (12XDUX4-luc) (Fig. 2A). As expected from previous work (Geng et al., 2011; Homma et al., 2015; Zhang et al., 2016), we found that the 12X DUX4 promoter was activated by DUX4-FL but was not activated by DUX4-S (which lacks the C-terminal TAD) (Fig. 2B).

The 12X DUX4 promoter was also activated by all constructs that had two intact DUX4 homeodomains coupled with amino acids from the DC2 C-terminal region (Fig. 1B), though the extent of activation differed depending on C-terminal amino acids included in the construct. Only one construct, delMid (equivalent to S+DC1+DC2 or S+344-424), activated the reporter to the same extent as DUX4-FL, whereas del405-424 and S+VP16 activated to ∼40-50% the level of DUX4-FL. S+DC2 (equivalent to S+375-424) and S+398-424 also produced low levels of activation at ∼10-25% the effect of DUX4-FL.

In contrast, the promoter was not activated by three constructs (delDC1/2, delDC2, and S+DC1) that completely lacked the most C-terminal DC2 region. Another construct (S+375-397), in which the most N-terminal half of the DC2 region was fused to DUX4-S but the C-terminal half of DC2 was missing, also failed to activate the promoter. In addition, all constructs with homeodomain mutations (HOX1, HOX2, HOX1/2, delMidHOX1/2) failed to activate the 12X DUX4 reporter above the vector control. Though the HOX1 mutant produced a small signal, this signal did not differ from control (*P*>0.1). The PAX3-DUX4 fusion produced a small signal that did not differ from control (*P*>0.1).

Expression of each construct produced a protein that localized to nuclei (Fig. 1C and not shown), which is consistent with the presence of multiple, widely distributed nuclear localization sequences within the DUX4 protein as found by Corona et al. (2013). Thus, lack of promoter activation was not due to exclusion from the nucleus.

**Fig. 3.**
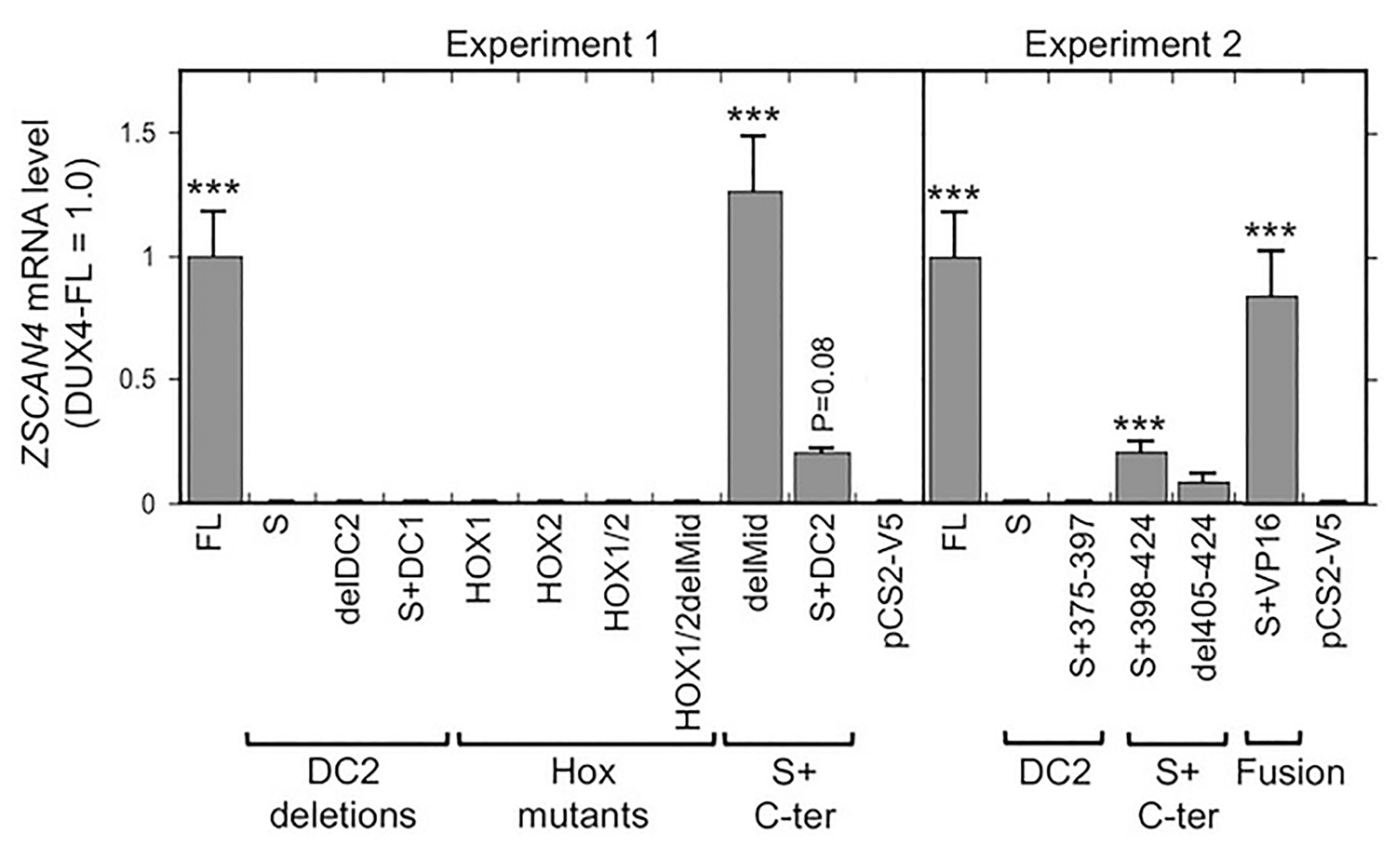
**Expression level of the DUX4-FL target *ZSCAN4* mRNA induced by DUX4 deletion and fusion proteins.** In two separate experiments, the level of *ZSCAN4* mRNA in HeLa cells was determined by real-time PCR as described in the Materials and Methods at 48 h after transfection of the indicated DUX4 deletion and fusion constructs. Expression of *ZSCAN4* was increased by intact DUX4-FL (FL) and, to different extents, by S+C-term constructs and the S+VP16 fusion protein. In contrast, expression of *ZSCAN4* was not increased by DUX4-S (S), S+374-397 or any of the Hox mutants. ***P<0.001 compared to vector control (pCS2-V5) by the Dunnett method; means±s.e.; *n*=3 for experiment 1; *n*=6 for experiment 2.

We next used RT-PCR to determine if expression of the endogenous *ZSCAN4* mRNA was altered by expression in HeLa cells of each of the DUX4 constructs. *ZSCAN4* is a well-characterized DUX4-FL target gene so that its mRNA expression level is a marker of DUX4 activity (Yao et al., 2014). For each construct, we typically found a close correlation between the *ZSCAN4* mRNA level (Fig. 3) and the level of activation of the 12X DUX4 promoter (Fig. 2). In particular, expression of DUX4-FL and delMid, i.e. the constructs with two intact homeodomains and the entire DC1+DC2 region, generated the largest increases in *ZSCAN4* mRNA levels. Moderate or low increases in *ZSCAN4* mRNA levels were generated by constructs with the two intact homeodomains combined with either a heterologous TAD (S+VP16) or with the entire or partial DC2 domain (S+DC2, S+398-424, and del405-424). Homeodomain mutants and constructs with complete DC2 deletions had no effect on *ZSCAN4* mRNA levels.

**Fig. 4.**
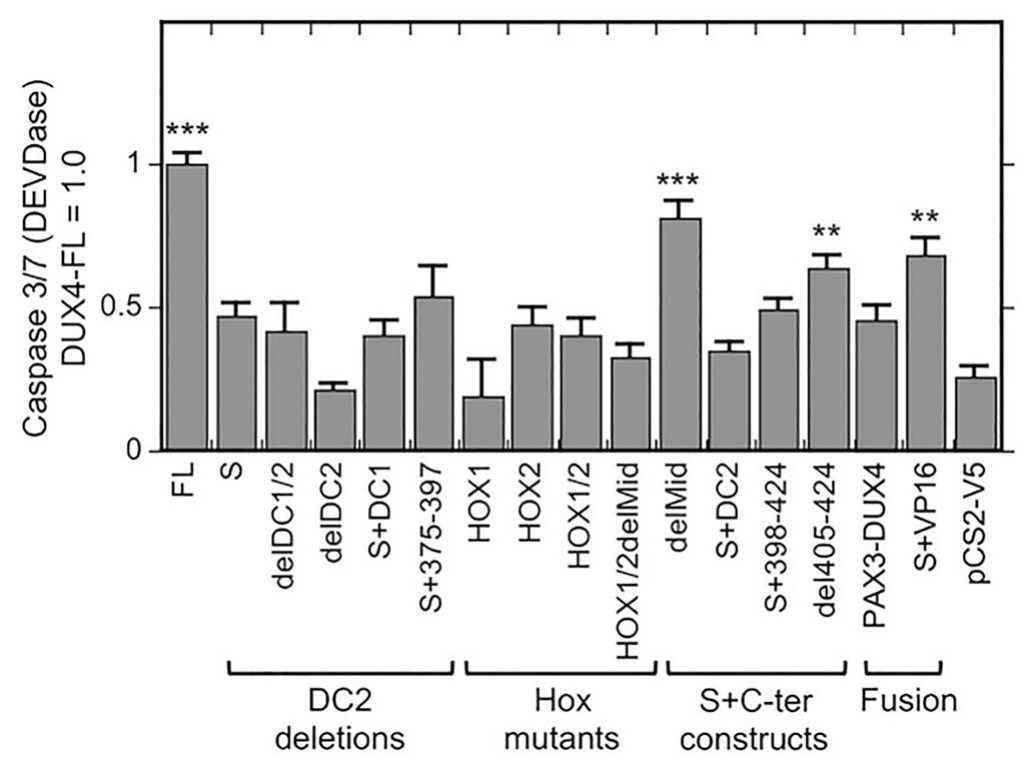
**Caspase 3/7 (DEVDase) activation by DUX4 deletion and fusion proteins.** HEK293 cells were transfected with the indicated DUX4 constructs, and caspase 3/7 (DEVDase) was determined as described in the Materials and Methods at 48 h after transfection. Caspase 3/7 (DEVDase) activity was increased by expression of intact DUX4-FL (FL) and, to different extents, by some S+C-terminal constructs and by the S+VP16 fusion protein. In contrast, caspase 3/7 (DEVDase) activity did not appear to be affected by expression of DUX4-S (S), the other DC2 deletions or any of the Hox mutants. ***P<0.001, **P<0.01 compared to pCS2-V5 control by the Dunnett method; means±s.e.; *n*=4.

**Fig. 5.**
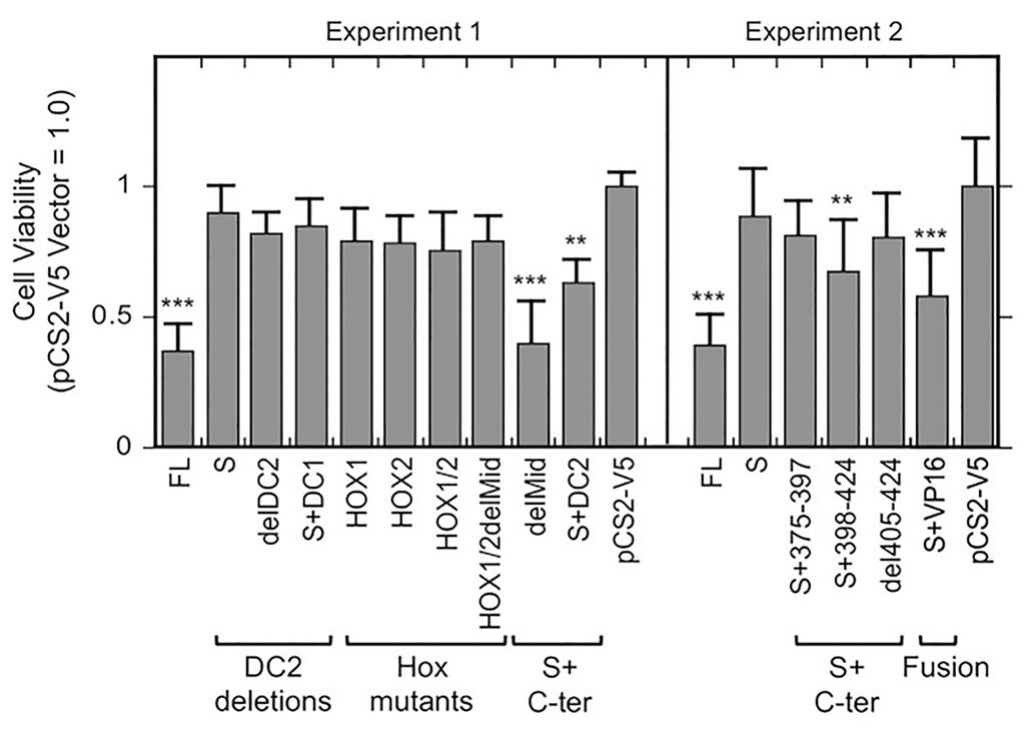
**Changes in cell viability induced by DUX4 deletion and fusion proteins.** In two separate experiments, HeLa cells were transfected with the indicated DUX4 constructs, and the number of viable cells was determined as described in the Materials and Methods at 48 h after transfection. The number of viable cells was decreased by expression of intact DUX4-FL (FL) and, to different extents, by some S+C-terminal constructs and the S+VP16 fusion protein. In contrast, cell viability did not appear to be affected by expression of DUX4-S (S), the other DC2 deletions or any of the Hox mutants. ***P<0.001, **P<0.01, compared to pCS2-V5 (vector) control by the Dunnett method; means±s.e.; *n*=3 for experiment 1; *n*=10 for experiment 2.

The DUX4 promoter and *ZSCAN4* mRNA assays both measured the ability of each DUX4 construct to directly activate transcription. To determine how transcription activation might correlate with cellular pathology, we next determined how expression of each construct affected activation of caspases 3/7 (i.e. DEVDase activity) (Fig. 4) and cell viability (Fig. 5). High-level activation of caspase-3 is a critical step in some cell death pathways and cell viability is a direct measure of toxicity.

For caspase activation assays, we transfected the DUX4 constructs into HEK293 cells and measured DEVDase activity at 48 h after transfection. We found that HEK293 cells had a measureable baseline level of DEVDase activity that was increased ∼3-4× by expression of DUX4-FL (Fig. 4). In addition to DUX4-FL, we found that expression of the delMid, S+VP16, and del405-424 constructs generated increased caspase activity at *P*≤0.01. These constructs were also active in the 12X promoter and *ZSCAN4* assays. Two constructs that had low activity in the 12X promoter and *ZSCAN4* assays, S+DC2 and S+398-424, did not raise caspase activity above baseline (i.e. *P*>0.1). All other tested constructs were also inactive in the caspase assay with *P*>0.1. Thus, results of the caspase activation assay were generally similar to the results of transcription assays, though the caspase assay had higher variability and a lower signal to background ratio than the transcription assays.

For cell viability assays, we transfected the DUX4 constructs into HeLa cells and used a colorimetric dye conversion assay to measure the extent of cell survival at 48 h after transfection (Fig. 5). In addition to DUX4-FL, we found that expression of the delMid, S+DC2, S+VP16, and S+398-424 constructs decreased the number of viable cells (i.e. caused cell death) at *P*≤0.01. All of these constructs were also active in the 12X promoter and *ZSCAN4* assays; and the FL, delMid, and S+VP16 constructs were active in the caspase activation assay. The del405-424 construct did not appear to affect cell viability (*P*>0.1), though this construct did have low activity (though at *P*<0.01) in the 12X promoter and caspase activation assays, as well as a low signal (though at *P*>0.1) in the *ZSCAN4* mRNA assay. All other tested constructs were also inactive in the cell viability assay with *P*>0.1. The results of the cell viability assay were generally similar to the results of the transcription and caspase activation assays, though the cell viability assay, similar to the caspase assay, had higher variability and a lower signal to background ratio than the transcription assays.

**Fig. 6.**
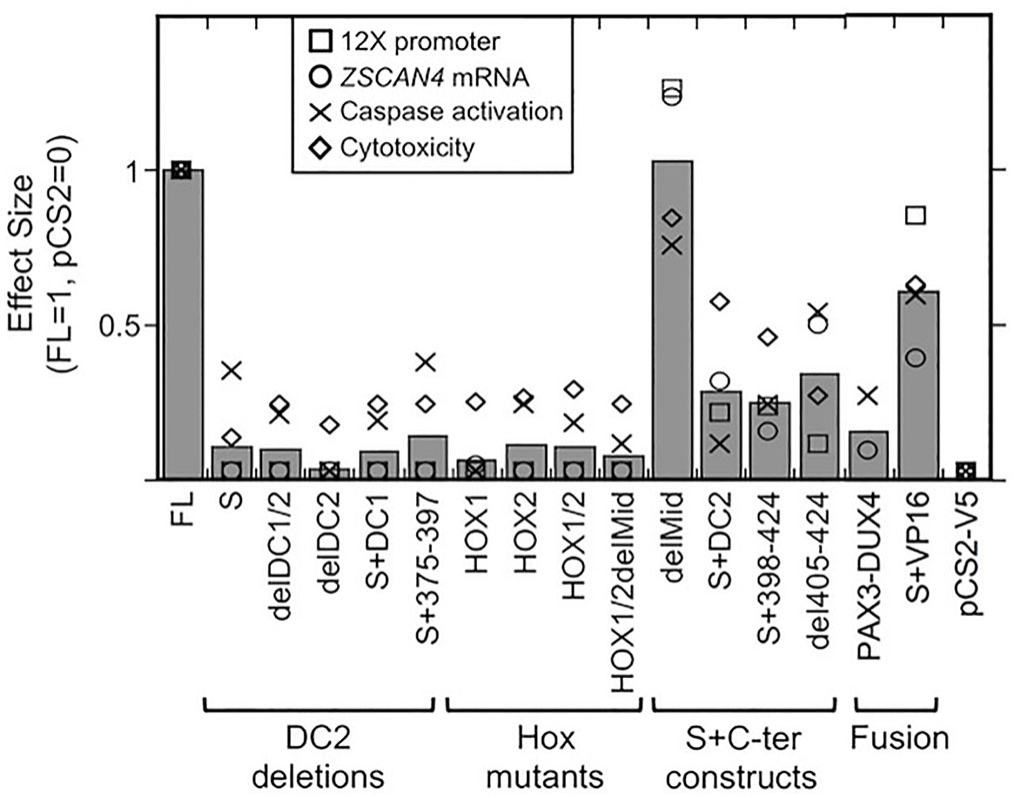
**DUX4 mutants produced relatively consistent effects across multiple assays.** Graph shows the effect size for each construct in four different assays and the average effect size. Values are normalized so that DUX4-FL=1 and pCS2(+)-V5=0. The DUX4-FL, delMid, and S+VP16 constructs consistently showed the largest effects; whereas the DUX4-S construct, the constructs with C-terminal deletions, and the homeobox mutants consistently showed the lowest effects. Intermediate effects were shown by the S+DC2, S+398-424, and del405-424 constructs. Gray bars=average value; □=12XDUX4-luc activation from Fig. 2; ○=*ZSCAN4* mRNA level from Fig. 3; ×=caspase activation from Fig. 4; ⋄=cytotoxicity from Fig. 5.

The results of the 12X DUX4 promoter, *ZSCAN4* mRNA, caspase activation, and cell viability assays are summarized in Fig. 6, which shows that the results for each construct were similar in each of the four assays. In particular, in all four assays, the greatest responses were generated by intact DUX4-FL and the delMid construct (which is equivalent to S+DC1+DC2). The next most effective construct was S+VP16, which also produced a positive response in each of the four assays, though typically at about half the extent of the signals generated by DUX4-FL and delMid. Constructs that were consistently ineffective in all four assays included DUX4-S, all of the single and double homeodomain mutants, and the constructs with the entire DC2 or the C-terminal-most half of the DC2 region deleted (i.e. S+DC1, delDC1/2, delDC2, S+375-397). Finally, a group of constructs showed low to moderate signals in each assay, sometimes, but not in each case, reaching *P*<0.01. The constructs in this group included (i) S+DC2, which included both homeodomains and amino acids 375-424; (ii) S+398-424; and (iii) del405-424, which included the entire DUX4-FL protein except for the most C-terminal 20 amino acids.

**Fig. 7.**
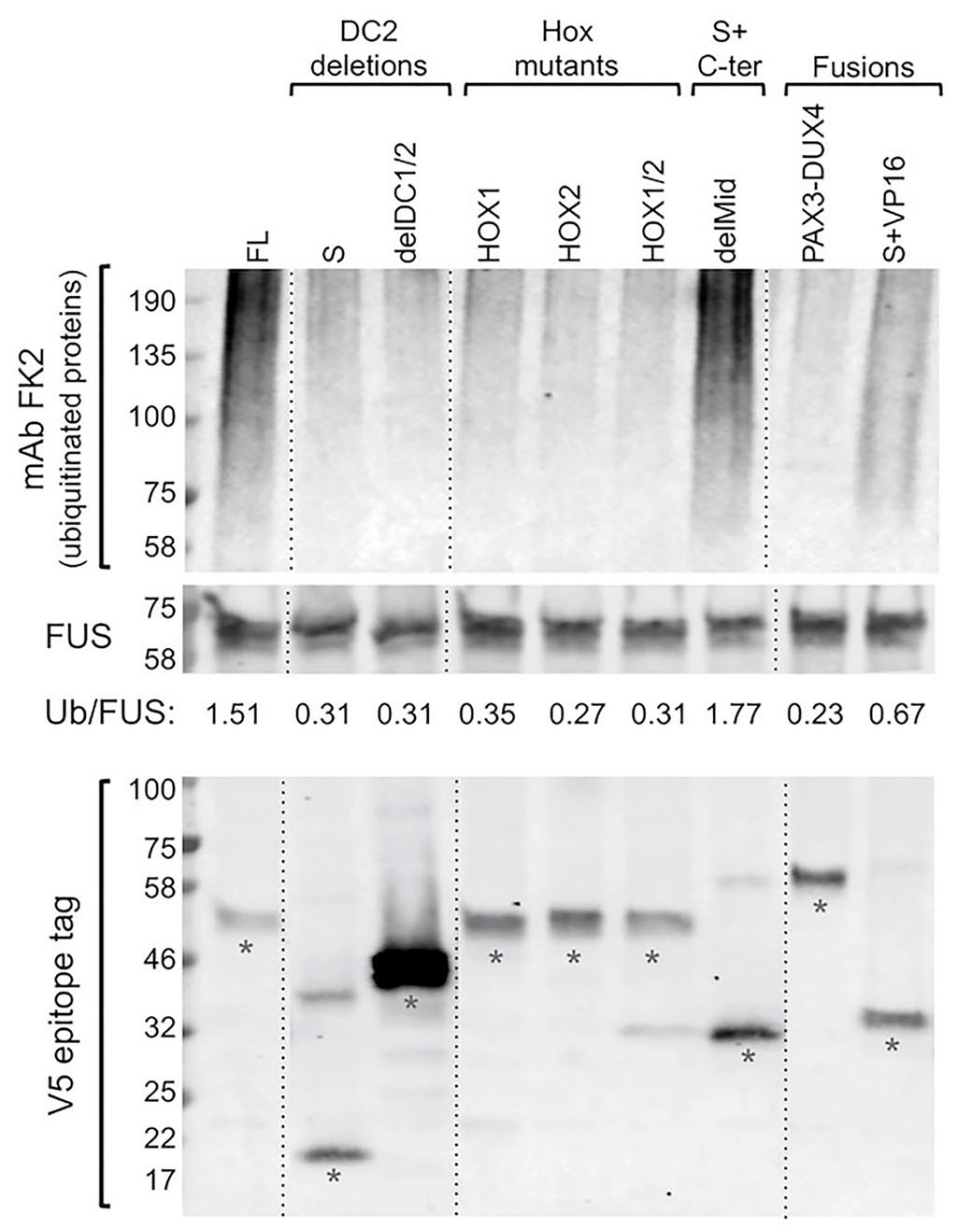
**Increased protein ubiquitination induced by DUX4-FL, delMid, and S+VP16 proteins.** Upper panel: HEK293 cells were transfected with the indicated DUX4 constructs, and the level of protein ubiquitination was determined by immunoblotting with mAb FK2 at 48 h after transfection. Middle panel: immunostaining for the protein FUS, which was unaffected by the transfected plasmids, served as a loading control. The ratio of ubiquitinated proteins to FUS (Ub/FUS) was determined by densitometry with ImageJ. As in our previous work (Homma et al., 2015), expression of DUX4-FL (FL), but not DUX4-S (S), increased the level of protein ubiquitination. Ubiquitination was also increased by expression of the delMid protein and, to a lesser extent, by the S+VP16 fusion protein, whereas ubiquitination was not affected by the other tested constructs. Thus, the three proteins with greatest effect in other assays (see Fig. 6) also had the greatest effect on protein ubiquitination. Lower panel: an immunoblot of the V5-tagged proteins produced from each transfected plasmid showed that each construct produced a major band (denoted by asterisks) that was of the appropriate predicted size. Though most of the tested constructs generated about the same level of V5-tagged protein (indicating that lack of effect on ubiquitination was not due to lack of expression), the delDC1/DC2 construct generated more protein than the other constructs, a result consistent with the finding of Bosnakovski et al. (2017a) that deletion of C-terminal regions increases DUX4 accumulation in transfected cells. All samples in each panel were from the same blot, but lanes were re-arranged (as indicated by the dotted lines) for presentation.

We next tested a group of the constructs for their ability to increase protein ubiquitination, as shown for DUX4-FL in our previous study (Homma et al., 2015). We found that expression of DUX4-FL, delMid, and S+VP16 – the three constructs that were most active in the previous assays – also increased the level of ubiquitinated proteins in HEK293 cells (Fig. 7). As in the previous assays, DUX4-FL and delMid generated the highest responses, whereas S+VP16 generated a smaller response. Also consistent with the previous assays, ubiquitination was not increased by the homeodomain mutants or by constructs that lacked the DC2 region. Immunoblots of the V5-tagged proteins (Fig. 7, lower panel) produced by each construct showed that each construct produced a major band that was of the appropriate predicted size. Though most of the tested constructs generated about the same level of V5-tagged protein (indicating that lack of effect on ubiquitination was not due to lack of expression), the delDC1/DC2 construct generated more protein than the other constructs. This result is consistent with the finding of Bosnakovski et al. (2017a) that deletion of C-terminal regions increases DUX4 accumulation in transfected cells. This study showed that ability of a construct to increase ubiquitination appeared to be correlated with its ability to act as a transcription factor.

**Fig. 8.**
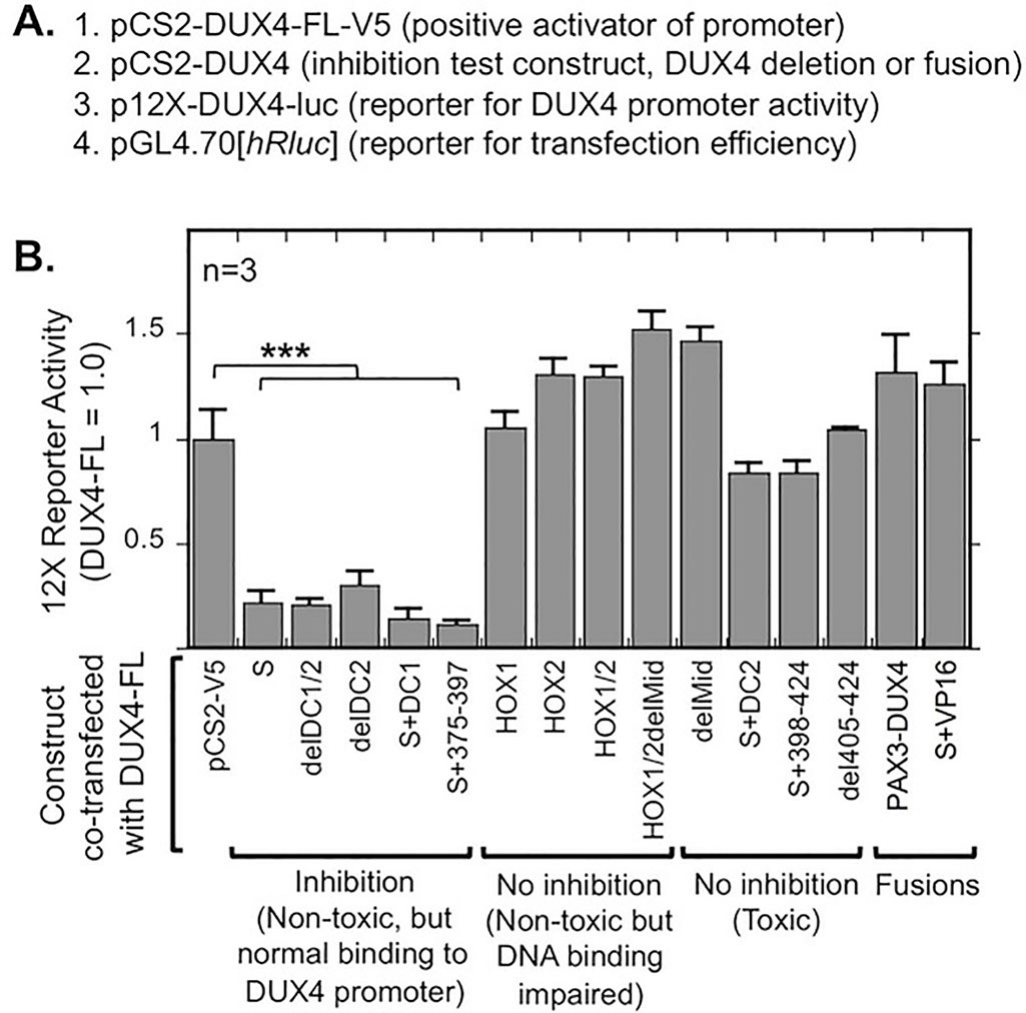
**DUX4 deletion and fusion constructs that were non-toxic and had intact homeodomains were dominant-negative inhibitors of DUX4-FL.** (A) For this experiment, four plasmids were co-transfected into HEK293 cells including (i) pCS2-DUX4-FL-V5, which is a positive activator of the 12X reporter, (ii) the DUX4 deletion or fusion construct that was to be tested for generation of a dominant-negative inhibitor, (iii) the 12X-DUX4 promoter-Luciferase reporter, to measure DUX4 promoter binding and subsequent activation of the luciferase reporter gene, and (iv) a *Renilla* luciferase reporter, to measure transfection efficiency for use in normalization. (B) As indicated, activation by DUX4-FL of the 12X promoter was inhibited only by those constructs that had two intact homeodomains and were non-toxic in other assays (see Figs. 2-7). HEK293 cells were transfected with a 1:3 ratio of DUX4-FL to test plasmid, and activity of the 12X reporter was measured 24 h after transfection. ***P<0.001 compared to pCS2-V5 control by the Dunnett method; means±s.e.; *n*=4.

In a final set of experiments, we examined the mechanism underlying the ability of DUX4-S to act as a dominant-negative inhibitor of DUX4-FL (Mitsuhashi et al., 2013; Snider et al., 2010). We tested the ability of each construct to inhibit DUX4-FL activation of the 12X DUX4 promoter-luciferase reporter by assaying reporter activity at 48 h after co-transfecting DUX4-FL and the test construct at a 1:3 ratio in HEK293 cells (Fig. 8). We carried out the assay with low amounts of transfected plasmids so that reporter activity would not be limited by competition for or sequestration of general transcription factors. The results showed DUX4-FL was inhibited only by those constructs that had two intact homeodomains but were themselves inactive in the 12X DUX4 promoter assay. These inhibitory constructs included DUX4-S, delDC1/2, delDC2, S+DC1, and S+375-397. In contrast, none of the single or double homeodomain mutants were able to inhibit DUX4-FL activation of the 12X promoter. Finally, co-transfections of DUX4-FL with those constructs that were able to activate the 12X promoter in single transfections (i.e. toxic constructs) (Fig. 2B) generated signals approximately the same size as those generated by DUX4-FL alone.

## DISCUSSION

In this study, we generated a series of DUX4 mutant, deletion, and fusion constructs and determined how expression of each of these constructs affected DUX4-induced changes in cellular and molecular properties that may be linked to FSHD pathogenesis. The results showed that each construct had similar effects in each of the assays we used, i.e. activation of the 12X DUX4 promoter reporter, level of endogenous *ZSCAN4* mRNA, caspase activation, cytotoxicity, and protein ubiquitination. Thus, the extent of change in multiple molecular and cellular properties was correlated with the ability to bind to and activate the 12X DUX4 promoter. In addition, to act as an inhibitor of DUX4-FL, a construct had to be itself non-toxic and to have both homeodomains intact, suggesting that inhibition was due to direct competition for promoter binding sites.

All of the constructs we produced (Table 1) localized to the nucleus, a finding that is consistent with the previous finding that the DUX4-FL protein has multiple, redundant sequences that mediate nuclear import (Corona et al., 2013). The three regions identified in that study – RRRR at amino acids 20-23, RRKR at amino acids 95-98, and RRAR at amino acids 145-148 – were not modified in any of our constructs. These authors also found a domain ‘around amino acids 314-338’ (in what we termed the disordered Mid region) that can contribute to nuclear localization when all three of the N-terminal localization motifs are mutated, but our constructs were not modified in a way to confirm that observation. Corona et al. (2013) further found that deletion of homeobox IWF sequences (amino acids 63-65 and 138-140) did not prevent nuclear localization; and, consistent with that observation, we found that our alanine-substitution mutations in the homeodomain IWF sequences also did not prevent nuclear localization.

We found that only one of our constructs – delMid – was consistently as active as DUX4-FL itself in each of our assays. In the delMid construct, the region from amino acid 160 through amino acid 343 was deleted so the resulting protein lacked most of the disordered Mid region and was equivalent to S+DC1+DC2 or S+344-424. Because delMid was as active as DUX4-FL, it appears that the disordered Mid region does not play a significant role in regulating DUX4 transcriptional activity or cytotoxicity, a conclusion also reached by Choi et al. (2016a). The full activity of the delMid construct also shows that the 81 most C-terminal amino acids of DUX4-FL (i.e. DC1+DC2) were sufficient to form a fully active TAD. A predicted 9aaTAD (classified as a 92% match by the prediction algorithm) is located at amino acids 371-379 of DUX4-FL, i.e. exactly spanning the boundary at amino acids 374-375 between the DC1 and DC2 regions as used in our constructs. Additional work will be needed to test whether this potential 9aaTAD is functional in DUX4-FL-regulated transcription, but our study and previous studies (Bosnakovski et al., 2017a) show that only constructs that include this predicted 9aaTAD are as active as DUX4-FL in cytotoxicity and transcription assays.

Our S+VP16 construct, in which amino acids 160-424 (i.e. the Mid, DC1, and DC2 regions) of DUX4-FL were replaced with the well-characterized VP16 TAD, was consistently about half as active as DUX4-FL and delMid in our assays. When fused to DUX4-S, therefore, the DC1+DC2 region (amino acids 344-424 of DUX4-FL) generated a stronger transcriptional activator in our assay than did the similarly sized VP16 TAD, even though VP16 is usually found to be a very strong activator (Hirai et al., 2010). In a previous study, Banerji et al. (2015) generated a fusion protein that included amino acids 1-350 of DUX4-FL fused to the VP16 TAD. When transfected into mouse myoblasts, that longer DUX4-VP16 fusion protein activated a transcriptional program that was similar to, but distinct from, the program activated by DUX4-FL, as determined from examination of microarray data by hierarchical clustering and principal component analyses. Thus, the DNA-binding specificity of the homeodomains, e.g. as identified by Zhang et al. (2016) and used in the 12X DUX4 promoter-luciferase reporter, determines which genes can be activated by DUX4-FL (Yao et al., 2014; Zhang et al., 2016). However, our results and those of Banerji et al. (2015) are also consistent with the possibility that the C-terminal DC1+DC2 region of DUX4-FL may function in determining the extent of a target gene's activation and/or whether particular genes with DUX4 binding sites are activated. Additional work is needed to test these ideas.

We found two types of constructs that were consistently inactive in our assays: homeodomain mutants and DC2 deletions. Homeodomain mutants, whether in homeodomain 1, homeodomain 2, or both, did not show activity different from vector controls in any of our assays. This result is consistent with previous studies that identified both intact homeodomains as required for DUX4 activity in transcription and cytotoxicity assays (Bosnakovski et al., 2017a; Corona et al., 2013; Mitsuhashi et al., 2013; Wallace et al., 2011). Also inactive in our assays (i.e. *P*>0.1) were constructs that lacked the entire DC2 region (DUX4-S, delDC1/DC2, delDC2, and S+DC1), as well as the construct (S+375-398) that lacked the C-terminal-most half of DC2. In a previous study (Bosnakovski et al., 2017a), a construct containing amino acids 1-399 of DUX4-FL was found to have low activity (e.g. ∼10-25% of DUX4-FL's activity in cytotoxicity, annexin V, and EDU incorporation assays). Both our study of S+375-398 and that of Bosnakovski et al. (2017a) with DUX4(1-399) support the idea that the C-terminal-most ∼25 amino acids of DUX4-FL are needed to generate the greatest toxicity. Previous studies also identified the C-terminus as necessary for DUX4-induced toxicity and as the region containing the TAD (Bosnakovski et al., 2008a; Choi et al., 2016b; Corona et al., 2013; Geng et al., 2012). In addition, analysis of CIC-DUX4 fusions in specific sarcomas show that fusion with the C-terminal 80 amino acids from 4q35-encoded DUX4-FL is sufficient to convert CIC into a transcriptional activator (Italiano et al., 2012; Kawamura-Saito et al., 2006).

Three of our constructs – S+DC2, S+398-424, and del405-424 (equivalent to DUX4 amino acids 1-404) – were only partially active in our assays. Though these constructs consistently produced positive signals, their effects were <25% of the effects induced by DUX4-FL and only sometimes at *P*<0.01. Because the delMid construct (equivalent to S+DC1+DC2 or S+344-424) had full activity, but S+DC2 (equivalent to S+375-424) had only partial activity, it appears that amino acids 344-374 (i.e. the DC1 region) likely play a role, in combination with DC2, in determining DUX4-FL activity. This conclusion is supported by a previous study (Bosnakovski et al., 2017a), which showed that full activity was reconstituted with the most C-terminal 98 amino acids (i.e. 327-424) of DUX4-FL, but not with the C-terminal-most 53 amino acids (i.e. 372-424). The most C-terminal 20 amino acids 405-424 similarly play a large role in determining activity, as shown by the reduced activity of the del405-424 construct compared to DUX4-FL or delMid. The C-terminal region of DUX4-FL is rich in acidic amino acids, e.g. eight of the last 25 amino acids are glutamic acids, but further work is needed to explore the roles of individual amino acids in forming the transcription activation domain(s). Taken together, the results suggest that the full transactivation activity of DUX4-FL requires multiple domains within the most C-terminal ∼80 amino acids, perhaps including a 9aaTAD and glutamic acid-rich regions.

Previous studies had shown that DUX4-FL-induced gene expression and cytotoxicity could be rescued by co-expression of DUX4-S (Mitsuhashi et al., 2013; Snider et al., 2010), which is the 159 amino acid short isoform of DUX4 that includes both homeodomains but lacks the disordered Mid region and the entire C-terminal transactivating region. To further analyze the mechanism underlying DUX4-FL inhibition, we used co-expression studies to identify which DUX4 domains were required to inhibit DUX4-FL-induced activation of the 12X DUX4-luc reporter of Zhang et al. (2016). We found that the presence of one mutated homeodomain was sufficient to prevent inhibition, a result that is consistent with a mechanism of inhibition that requires both homeodomains to be intact to effectively compete directly with DUX4-FL for promoter sites.

At least two additional mechanisms for inhibition of DUX4 might be considered, though both seem less likely than direct competition for promoter sites. One alternative mechanism is that the inhibitory constructs interacted with DUX4-FL to generate inactive dimers (or higher order multimers). Because our homeodomain mutants failed to act as inhibitors, however, this mechanism would require multimerization to be prevented by each of the two individual homeodomain mutations that we generated. Though proteins that have only one homeodomain, e.g. PITX2 (Saadi et al., 2003), can form dimers, there is so far no clear evidence that DUX4-FL forms homodimers. With its two homeodomains, DUX4-FL may be functionally equivalent to a dimer as suggested by the finding that DUX4 is 20× more active on a reporter with two DNA binding sites than one (Zhang et al., 2016). Another alternative mechanism is that the inhibitory constructs might have bound to and sequestered cofactors, including general transcription factors, which were needed for DUX4-FL function. This mechanism seems unlikely both because of the low amounts of plasmids used and the modest 3× higher expression of the test constructs compared to DUX4-FL and because mutation in either homeodomain would have to be sufficient to prevent sequestration. Though direct competition for promoter sites is the simplest explanation for our competition results, we cannot definitively eliminate the alternative mechanisms so further investigation is warranted.

Though our work showed that several markers of pathology appear to be determined by DUX4-FL transcription activity, that conclusion may not hold for all of the potentially pathological functions that have been attributed to DUX4-FL. In particular, the DUX4(1-217) construct, which contains both homeodomains but lacks most of the Mid region and all of the C-terminal TADs, inhibits myotube formation when expressed in mouse C_2_C_12_ myoblasts (Bosnakovski et al., 2017a), perhaps due to competition with PAX3 and/or PAX7. In addition, DUX4-FL alters splicing patterns and expression of multiple genes in addition to *ZSCAN4* (Banerji et al., 2017; Jagannathan et al., 2016; Rickard et al., 2015), but we did not determine how our constructs affected larger patterns of gene expression. Also remaining to be determined is whether transcriptional activity correlates with DUX4-FL-induced nuclear aggregation of TDP-43, FUS, and SC35 (Homma et al., 2015, 2016), with DUX4-FL-mediated accumulation of dsRNA and nuclear aggregation of EIF4A3 (Shadle et al., 2017), or with inhibition of nonsense-mediated decay (Feng et al., 2015). To develop therapies for FSHD, several groups are developing techniques to genetically or pharmacologically inhibit the function or expression of DUX4-FL (Ansseau et al., 2017; Bosnakovski et al., 2014; Campbell et al., 2017; Chen et al., 2016; Choi et al., 2016b; Himeda et al., 2016; Lim et al., 2015; Peart and Wagner, 2017; Rickard et al., 2015; Teveroni et al., 2017; Wallace et al., 2012, 2017). Because multiple downstream pathological changes are correlated with DUX4-FL transcriptional activity, any strategy that inhibits DUX4-FL expression or function should prevent additional pathology – that is dependent on DUX4-FL transcriptional activity – from occurring after the onset of treatment.

## CONCLUSIONS

Each of the DUX4 mutant, deletion, and fusion constructs produced similar effects in each of the assays we used, i.e. activation of the 12X DUX4 promoter reporter, level of endogenous *ZSCAN4* mRNA, caspase activation, cytotoxicity, and protein ubiquitination. Thus, the ability to activate transcription was correlated with the extent of change in multiple molecular and cellular properties that may be relevant to FSHD pathology. Transcriptional activity was mediated by the C-terminal 80 amino acids of DUX4-FL, with most activity dependent on the most C-terminal 20 amino acids. In addition, to act as an inhibitor of DUX4-FL, a construct had to be itself non-toxic and to have both homeodomains intact, suggesting that inhibition was most likely due to direct competition for promoter binding sites.

## MATERIALS AND METHODS

### Antibodies

Rabbit anti-DUX4-FL mAb E55 which reacts with a C-terminal domain epitope (Geng et al., 2011) was used at 1:200 dilution (cat. ab124699, Abcam). GAPDH was detected with a mouse mAb (cat. 10R-G109A, Fitzgerald, Acton, USA) used at 1:5000 dilution. The V5 epitope tag was detected using either mouse anti-V5 mAb (cat. R960-25, Thermo Fisher Scientific) used at 1:500 or a rabbit pAb (cat. AB3792, EMD Millipore) used at 1:300. Ubiquitinated proteins were detected with mouse mAb FK2 (cat. D058-3, MBL International, Woburn, USA) used at 1:1000; FK2 reacts with K29, K48, and K63 mono- and poly-ubiquitinated proteins, but not with free ubiquitin. FUS was detected with a rabbit pAb (cat. 11570-1-AP, lot 00024677; ProteinTech, Rosemont, USA) used at 1:200. Each of the primary antibodies was validated based on one or more methods, including prior use in multiple published studies with the same mAb or lot of polyclonal antiserum, manufacturer's validation assays including knockouts, generation of expected immunofluorescence staining patterns, detection of appropriate band size on immunoblots without detection of non-specific bands, and detection of recombinant protein when expressed in cells that normally do not express the protein.

### Cells and culture

Cells of the human HeLa line were obtained from the RIKEN BRC Cell Bank (cat. RCB0007, Tsukuba, Japan); and cells of the human embryonic kidney line 293 (HEK293) were obtained from the American Type Culture Collection, Manassas, USA (cat. CRL1573). HEK293 and HeLa cells were grown in Minimal Eagle's Medium (cat. M2279, Sigma-Aldrich) or Dulbecco's Modified Eagle's Medium (cat. D5796, Sigma-Aldrich) supplemented with 10% fetal bovine serum (cat. 10270-106, Thermo-Fisher Scientific; or cat. SH30070, HyClone GE Life Sciences, USA).

### DUX4-FL domain predictions

We used several internet-based prediction sites to identify likely structural features of the endogenous DUX4-FL protein. Sites that we consulted include RaptorX at http://raptorx.uchicago.edu/StructurePrediction/ (Källberg et al., 2012) (Fig. 1A); MetaDisorder at http://genesilico.pl/metadisorder/ (Kozlowski and Bujnicki, 2012); Phyre^2^ at http://www.sbg.bio.ic.ac.uk/~phyre2 (Kelley et al., 2015); Robetta at http://robetta.bakerlab.org (Song et al., 2013); and the Eukaryotic Linear Motif Resource at http://elm.eu.org (Dinkel et al., 2016). Each of these sites similarly predicted that regions with well-defined tertiary structure in DUX4-FL would be limited to the two homeodomains and a C-terminal domain and that the long ‘Mid’ region between the second homeodomain and the C-terminal domain would be disordered (Fig. 1A and not shown). We used an additional prediction tool at http://www.med.muni.cz/9aaTAD/ (Piskacek et al., 2016) to identify a potential nine amino acid transactivation domain (9aaTAD) in DUX4-FL (Fig. 1A).

### DNA constructs

The pCS2(+)-V5 host vector was prepared as described previously (Mitsuhashi et al., 2013). A diagram of the DUX4 protein with relevant features is shown in Fig. 1B, and descriptions of the constructs used in this study are given in Table 1. The NCBI reference sequence for the full-length DUX4 protein is NP_001292997.1 and this sequence is shown in Fig. 1C as modified by the linker plus V5 epitope sequence that was added to the C-terminal end of every construct described in Table 1.

The human DUX4-fl and DUX4-s cDNAs were cloned as previously reported (Mitsuhashi et al., 2013).

The HOX1 mutant, in which the WFQNER sequence beginning at amino acid number 66 was altered to AAQAAA, was generated as described previously (Mitsuhashi et al., 2013).

The HOX2 and HOX1/2 mutants were generated by site-directed mutagenesis with primers 1 and 2 (all primers are shown in Table 2) using the DUX4-fl and HOX1 mutants as templates, respectively. In the HOX2 mutant, the WFQNRR sequence beginning at amino acid 141 was converted to AAQAAA.

**Table 2.**
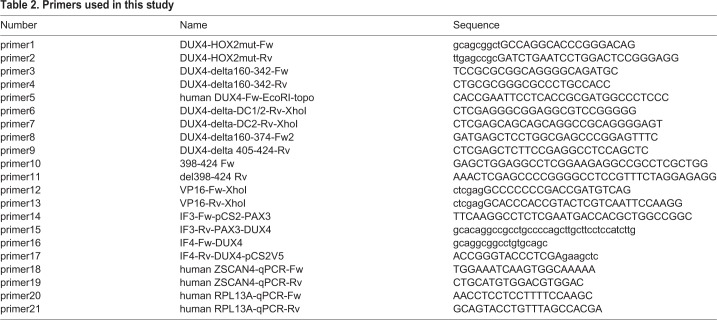
**Primers used in this study**

The delMid, delDC1/2, delDC2, S+DC2, del405-424, and S+398-424 mutants were generated by PCR with a PrimeSTAR GXL DNA polymerase (TaKaRa, Shiga, Japan) using DUX4-fl as a template. The following primers were used: primers 3 and 4 for delMid, primers 5 and 6 for delDC1/2, primers 5 and 7 for delDC2, primers 4 and 8 for S+DC2, primers 5 and 9 for del405-424, and primers 4 and 10 for S+398-424, respectively.

The S+DC1 construct was amplified using delMid as a template with primers 5 and 7. The HOX1/2-delMid construct was amplified using HOX1/2 as a template with primers 3 and 4. The S+375-397 mutant was amplified using S+DC2 mutant as a template with primers 5 and 11. DUX4-S-VP16 was generated by insertion of the VP16 fragment amplified with pBT3-N and primers 12 and 13 into the XhoI site of DUX4-s_pCS2(+)-V5.

All the PCR fragments were cloned into pCR-blunt vector (Invitrogen, Carlsbad, USA), digested with EcoRI and XhoI, and subcloned into pCS2(+)-V5 (Mitsuhashi et al., 2013).

To generate the PAX3-DUX4_pCS2(+)-V5 construct, the N-terminus of human PAX3D ORF (nt. 1-837), including paired box and homeodomain, and the transcriptional activation domain of DUX4 (nt. 478-1272 of DUX4-fl ORF) were PCR amplified with primers 14 and 15, and 16 and 17, respectively. The PCR products were purified and mixed with pCS2(+)-V5 vector digested with XhoI, and then the DNA fragments were ligated with an In-Fusion HD cloning kit (TaKaRa, Mountain View, USA) according to the manufacturer's protocol.

The sequences of all constructs were verified by DNA sequencing with an ABI 3500xL Genetic Analyzer (Applied Biosystems, Foster City, USA).

### Transfection

The deletion, fusion, and mutated proteins (Table 1) were expressed under control of the simian CMV IE4 promoter derived from pCS2(+). Plasmids were transfected into HeLa or HEK293 cells using the X-treme GENE 9 HP DNA transfection reagent (cat. XTGHP-RO, Roche Diagnostics, Indianapolis, USA) diluted in Opti-MEM I (Gibco) following the manufacturer's instructions.

### Immunocytology

Transfected HeLa cells were fixed with 2% paraformaldehyde at room temperature for 10 min at 24 h after transfection and permeabilized with 1% TritonX-100 at 4°C for 15 min. Fixed cells were incubated with 2% BSA at 37°C for 30 min for blocking. Anti-V5 antibody (Thermo Fisher Scientific, 1:500) was added at 4°C overnight. Alexa 546 conjugated-anti-mouse IgG antibody (Thermo Fisher Scientific, 1:600) and 1 μg/ml of Hoechst 33342 (Sigma-Aldrich) were added at room temperature for 45 min. Fluorescence was observed with a fluorescent microscope BZ-9000 BIOREVO (KEYENCE, Osaka, Japan).

### Immunoblotting

Use of immunoblotting to analyze ubiquitin-conjugated proteins, FUS, and V5-tagged proteins was carried out as described previously (Homma et al., 2015). Immunoblots were quantified using the grey scale densitometric function of the NIH ImageJ software v.1.51 available at https://imagej.nih.gov/ij/download.html.

### 12X DUX4 promoter reporter assay

The p12X-DUX4-luc (reporter for DUX4 promoter activity) and pGL4.70(*hRluc*) (reporter for transfection efficiency) plasmids were gifts from Dr Michael Kyba and were described previously (Zhang et al., 2016). For promoter activation assays, HEK293 cells in 96-well plates were transfected simultaneously with (i) Renilla control plasmid at 20 ng/well, (ii) the 12XDUX4-luc reporter at 50 ng/well, and (iii) the DUX4-FL, control, or mutant expression plasmid at 50 ng/well. Luciferase activity was analyzed at 24 h after transfection. For competition assays, HEK293 cells in 96-well plates were transfected simultaneously with (i) Renilla control plasmid at 20 ng/well, (ii) the 12XDUX4-luc reporter at 50 ng/well, (iii) the DUX4-FL expression plasmid at 50 ng/well, and (iv) the mutant plasmid or control pCS2(+)-V5 plasmid at 150 ng/well, thus giving a 3:1 ratio of mutant to DUX4-FL. Luciferase activity was analyzed at 24 h after transfection by Dual-Glo Luciferase Assay System (cat.E2920, Promega, Madison, USA) according to the manufacturer's instructions.

### *ZSCAN4* mRNA assay

At 24 h after transfection of 2 µg of plasmid into HeLa cells on six-well plates, the cells were harvested and total RNA was extracted with the GenElute Mammalian Total RNA Miniprep Kit (cat. RTN10, Sigma-Aldrich) with DNase I treatment (Sigma-Aldrich). The first-strand cDNA was synthesized from 1 µg of total RNA of each sample using PrimeScript 1st strand cDNA Synthesis Kit (cat. 6110A, Takara Bio) with Oligo dT primer. The expression level of endogenous *ZSCAN4* mRNA in HeLa cells transfected with DUX4 constructs was quantified with 7500 Real-Time PCR Systems (Applied Biosystems). The *ZSCAN4* transcript was amplified with PowerUP SYBR Green PCR Master Mix (Applied Biosystems) using primers 18 and 19. The expression levels of each transcript were normalized to a housekeeping gene, *RPL13A*, which was amplified with primers 20 and 21. The *ZSCAN4* level was calculated with the comparative Ct method. Undetermined values were equated to zero. Standard deviations from the mean of the ΔCt values were calculated from triplicates. The primers amplified specific PCR products as confirmed by polyacrylamide gel electrophoresis.

### Caspase activity

DEVDase activity (i.e. Caspase 3 and 7) was measured using the Caspase-Glo 3/7 enzymatic assay kit (cat. G8090, Promega, Madison, USA). The Cell-titer Fluor assay kit (cat. G6080, Promega) was used to measure relative cell numbers. All enzyme assays were carried out according to manufacturer's instructions. To correct for differences in viable cell numbers, the results of the DEVDase assay (Caspase-Glo) for each construct was divided by the corresponding result of the viable cell assay (Cell-titer Fluor) to generate a caspase/cell number ratio. For presentation as noted in each figure, these ratios were normalized by designating either the value for DUX4-FL or the value for pCS2(+)-V5 equal to one and adjusting ratios for the other constructs accordingly.

### Cytotoxicity

HeLa cells were transfected with 2 µg of plasmids and viable cells were assayed at 48 h after transfection by microplate reader SH-9000 (CORONA electric) using Cell Counting Kit-8 (cat. CK04; Dojindo Molecular Technologies, Kumamoto, Japan), a colorimetric assay, according to the manufacturer's instructions.

### Statistics

Results were analyzed with the Dunnett test with alpha=0.1 against the vector control using either R software version 2.15.1 (http://www.r-project.org/) or GraphPad Prism 7 (GraphPad Software). All sample sizes (*n*) used for statistical tests and for figures were biological replicates, i.e. measurements from independent samples. Graphed points are means±s.e.m.

## Supplementary Material

First Person interview
