# Supplementary material for: Functional domains of the FSHD-associated DUX4 protein
Source: Biol Open. 2018 Apr 4;7(4):bio033977. doi: 10.1242/bio.033977 (PMC5936065; doi:10.1242/bio.033977)
Supplement: First Person interview [file biolopen-7-033977-s1.pdf]

FIRST PERSON

First person – Hiroaki Mitsuhashi

First Person is a series of interviews with the first authors of a selection of papers published in Biology Open, helping early-career researchers promote themselves alongside their papers. Hiroaki Mitsuhashi is first author on 'Functional domains of the FSHD-associated DUX4 protein', published in BiO. Hiroaki is a Junior Associate Professor and runs his own lab at Tokai University, Kanagawa, Japan, investigating the pathomechanisms and development of therapeutics for muscular dystrophies.

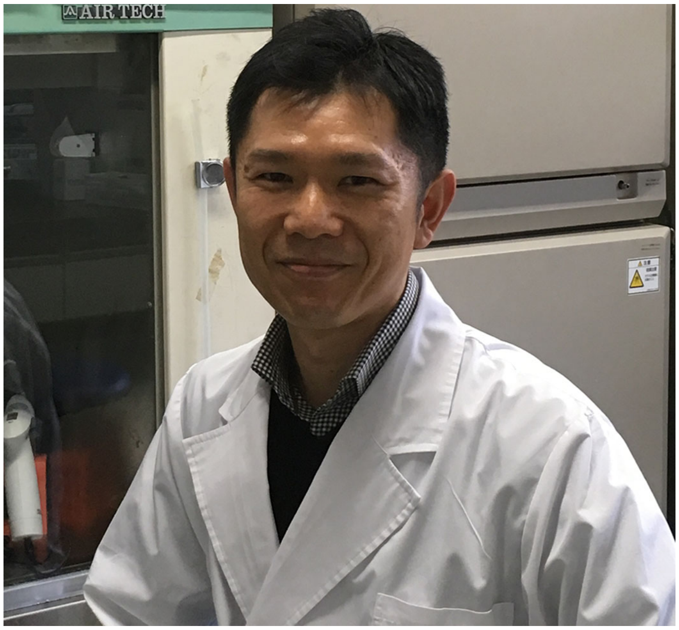

Hiroaki Mitsuhashi

What is your scientific background and the general focus of your lab?
I focused on molecular biology during my undergraduate studies. Aiming to develop potential therapeutics for muscular dystrophies, I launched my own laboratory at Tokai University in Japan three years ago, and I am investigating the pathomechanism of muscular dystrophies using human cell and zebrafish model systems.

How would you explain the main findings of your paper to non-scientific family and friends?
Facioscapulohumeral muscular dystrophy (FSHD) is a genetic disease that affects skeletal muscle, leading to muscle weakness and atrophy. There is currently no cure. Previous studies have revealed that FSHD is caused by abnormal synthesis of the DUX4-fl protein in muscles. In this paper, we closely examined which part of the DUX4-fl protein is responsible for its toxicity, and we identified that this region is at one end of the protein (the C-terminus). This result suggests the possibility that inhibiting DUX4-fl by targeting this specific region may ameliorate FSHD.

What are the potential implications of these results for your field of research?
DUX4-fl is a transcription factor. We demonstrated that its transcriptional activity is well-correlated with its cytotoxicity. Also, we revealed that its transcriptional activity depends on both intact homeodomains and the 80 C-terminal amino acid residues. These results suggest that inhibition of the transcriptional activity of DUX4-fl by targeting these specific regions is a rational approach to develop therapy for FSHD.

What has surprised you the most while conducting your research?
It was surprising that deletion of only 80 amino acid residues at the C-terminus eliminated the cytotoxicity and transcriptional activity of DUX4-fl. By contrast, fusion of the 80 amino acid residues to a non-toxic DUX4 isoform (DUX4-s) restored cytotoxicity and transcriptional activity.

What, in your opinion, are some of the greatest achievements in your field and how has this influenced your research?
The finding that specific genetic polymorphisms create a polyadenylation signal for the *DUX4* gene, thus allowing DUX4 transcript expression (Lemmers et al., 2010) had a great influence on our field. After their discovery, many researchers revealed the detrimental impact of DUX4 expression on muscle cells.

What changes do you think could improve the professional lives of early-career scientists?
There are not enough academic positions for young scientists, especially in Japan. Also, more grant opportunities for young scientists are necessary.

"I am interested in what can modify the transcriptional activity of DUX4-fl. Identifying such a mechanism would open the door to a new strategy to develop therapy for FSHD."

What's next for you?
I am interested in what can modify the transcriptional activity of DUX4-fl. Identifying such a mechanism would open the door to a new strategy to develop therapy for FSHD.

Hiroaki Mitsuhashi's contact details: Tokai University, School of Engineering, Department of Applied Biochemistry, 4-1-1 Kitakaname, Hiratsuka, Kanagawa 259-1292, Japan.
E-mail: hmitsuhashi@tsc.u-tokai.ac.jp

References
Lemmers, R. J. L. F., van der Vliet, P. J., Klooster, R., Sacconi, S., Camaño, P., Dauwerse, J. G., Snider, L., Straasheijm, K. R., van Ommen, G. J., Padberg, G. W. et al. (2010). A unifying genetic model for facioscapulohumeral muscular dystrophy. *Science* **329**, 1650-1653.
Mitsuhashi, H., Ishimaru, S., Homma, S., Yu, B., Honma, Y., Beermann, M. L. and Miller, J. B. (2018). Functional domains of the FSHD-associated DUX4 protein. *Biol. Open* **7**: bio033977.
